# High-level expression of biologically active human follicle stimulating hormone in the Chinese hamster ovary cell line by a pair of tricistronic and monocistronic vectors

**DOI:** 10.1371/journal.pone.0219434

**Published:** 2019-07-05

**Authors:** Nadezhda A. Orlova, Sergey V. Kovnir, Yulia A. Khodak, Mikhail A. Polzikov, Victoria A. Nikitina, Konstantin G. Skryabin, Ivan I. Vorobiev

**Affiliations:** 1 Laboratory of Mammalian Cell Bioengineering, Institute of Bioengineering, Research Center of Biotechnology of the Russian Academy of Sciences, Moscow, Russia; 2 IVFarma LLC, Moscow, Russia; 3 Laboratory of Radiation Hematology and Cytogenetics, State Research Center-Burnasyan Federal Medical Biophysical Center of Federal Medical Biological Agency, Moscow, Russia; 4 Laboratory of Biocatalysis, Institute of Bioorganic Chemistry of the Russian Academy of Sciences, Moscow, Russia; Instituto Butantan, BRAZIL

## Abstract

Recombinant human follicle stimulating hormone (FSH), produced in Chinese hamster ovary (CHO) cells, is widely used for treatment of fertility disorders and is subject to biosimilars development. Cell lines with high specific productivities may simplify the FSH production process. Here, we used our previously established expression system based on vector p1.1 to create new cell lines secreting heterodimeric FSH protein. To this end, we linked open reading frames of both FSH subunits by the wild-type internal ribosome entry site from the encephalomyocarditis virus (EMCV IRES). Intact and double-negative for the dihydrofolate reductase CHO cells were stably transfected by the FSH-coding plasmids. Stably transfected intact cells showed higher level of the FSH secretion and were utilized for subsequent methotrexate-driven transgene amplification, which doubled their productivity. The excess of the free α-subunit was corrected by transfecting the cells by the additional p1.1-based plasmid encoding the β-subunit of the FSH. Clonal cell lines obtained secreted mostly the heterodimeric FSH and possessed specific productivities up to 12.3±1.7 pg/cell/day. Candidate clonal cell line C-P1.3-FSH-G4 maintained a constant specific productivity for at least 2 months of culturing without the section pressure. The resulting FSH protein conformed to the international pharmaceutical quality criteria as evidenced by the receptor binding kinetics, distribution pattern of hormone isoforms and biological activity. In conclusion, our expression system offers a simple and cost-effective approach to production of FSH.

## Introduction

Follicle stimulating hormone (FSH) is a heterodimeric glycoprotein produced by the pituitary gland that promotes growth and maturation of ovarian follicles in females and spermatogenesis in males [1]. Urinary-derived and recombinant forms of FSH are widely used in infertility treatment for assisted reproducing technologies [2]. Since both of the above have the same efficacy and safety profiles, the clinical choice of FSH source is usually defined by availability, convenience, and cost [2]. As patents on innovative recombinant FSH products approach expiration, there is an increasing global interest in developing methodology for biosimilar FSH generation [3].

FSH consists of two dissimilar, non-covalently linked subunits with two sites for N-glycosylation at each polypeptide chain [4,5]; mature α-chain of the FSH consists of 96 aminoacids (10.3 kDa), mature β-chain is of 111 aminoacids (12.5 kDa). FSH is abundantly glycosylated and has an apparent molecular weight of about 34 kDa. Glycosylation plays an important role in its specific activity because affinities for the receptor and half-life *in vivo* for different isoforms vary in wide range. Namely, acidic hormone isoforms, containing more terminal sialic residues on the N-linked glycans, demonstrated lower affinity toward the FSH receptor and lower specific activity in *in vitro* tests, but show longer half-life in the bloodstream [6,7]. On the other hand, neutral FSH isoforms are as active as moderately acidic ones *in vitro* and have significantly decreased plasma half-life. The FSH preparations for clinical use should be devoid of non-sialylated variants and possess a proper balance between slightly acidic and more highly acidic isoforms [8]. Since bacterial and yeast expression systems cannot recapitulate the relevant glycosylation profile, the currently used expression systems for recombinant FSH production are based on mammalian cell lines, especially CHO cells [3]. The typical FSH production rate reported to date is 0.5–1.5 pg/cell/day [9–11]. Clonal CHO-based cell line, described as being able to produce a “highly expressed” FSH demonstrated a specific productivity of only 0.0085 mIU/cell/48 hours, which corresponds to 0.32 pg/cell/day level [12].

There are multiple ways of balancing the expression level of two genes encoding subunits of the heterodimeric protein. The simplest way is the co-transfection of two plasmids with different selection markers, each encoding one of the two polypeptide chains under the control of the same promoter [13]. In this case, successive target genes amplification in the genome of host cells, performed as the gradually increase of the selection pressure of the dihydrofolate (DHFR, EC 1.5.1.3) inhibitor methotrexate (MTX) is questionable. Many individual cells will amplify only one target gene, linked with the DHFR selection marker, leaving the second gene un-amplified and under-expressed. As the result, many individual cell clones should be obtained from the initial stably transfected cell population and screened for the ability to increase production of the heterodimeric protein after exposure to increasing MTX levels.

Another method consists in the construction of the single plasmid, containing both genes under control of two identical or different promoters. In this case, both genes may be MTX-amplified right in the polyclonal population of stably transformed cells, but relative levels of two genes expression will remain constant. This will result in the secretion of the mixture of target heterodimeric protein and one of its subunits in the monomeric form (i.e. free chain) [14]. Two genes may be linked in one expression plasmid by the IRES sequence instead of the promoter duplication. This design can provide the tightest link between two target genes and make the MTX-driven amplification more effective, although the resulting expression level of two genes still may be not balanced [15]. The second plasmid, coding the under-expressed chain with another selection marker may be transfected to the polyclonal MTX-amplified cell population and cell clones secreting mostly the heterodimeric protein may be picked up and used for target protein production.

Previously we have designed the set of vectors, based on endogenous translation elongation factor 1 α 1 (EEF1A) gene from Chinese hamster (*Cricetulus griseus*) and the fragment of long terminal repeats from Epstein-Barr virus, named p1.1 and p1.2 respectively [16, 17]. These vectors allow the high level of target genes expression in CHO cells at the relatively low insertion copy numbers. Also, unlike the initial EEF1A1-based vector pDEF38, the p1.1 vector yields much more stably transfected cell colonies at the initial selection stage due to the presence of the terminal repeat fragment from the Epstein-Barr virus [16]. The vector p1.1 utilizes the DHFR selection marker and is suitable for methotrexate-driven target gene amplification and p1.2 vectors with antibiotic resistance genes may be co-transfected for expression of additional genes. We used the p1.1 vector for development of cell lines, secreting human blood clotting factor VIII with the end-titer up to 40 IU/ml and utilized p1.1 and two p1.2 vectors for cell lines, secreting fully active blood clotting factor IX with the end-titer 6 IU/ml in the simple batch culturing [17]. In the latter case, p1.2 vectors with the zeocin and hygromycin selection markers were utilized to transfect supporting genes, coding enzymes VKORC1 and PACE/furin, which enabled the proper post-translational modifications of the factor IX molecule [18]. Both these genes were expected to express at lower levels than the target gene.

We hypothesized that in the case of heterodimeric protein, the vector pair consisting of one genome-amplifiable p1.1 vector and one non-amplifiable vector may be utilized for creation of cell lines with high specific productivity. The proper balance between expression levels of two polypeptide chains may be achieved if the genome-amplifiable p1.1 vector encodes both chains by the single multicistronic RNA and the second non-amplifiable vector encodes one chain, under-expressed by the first vector plasmid. Open reading frames of two chains may be interspersed by the wild-type IRES of the encephalomyocarditis virus (EMCV), this variant of IRES is known to provide the highest level of translation initiation [19]. In the p1.1 vector plasmid the target ORF is already linked to the mouse DHFR ORF by the attenuated EMCV IRES, so the resulting mRNA will be tricistronic, this order is expected to provide the tightest connection of both target ORFs with the selection marker ORF. This tight link may be more resistant to the MTX-driven genome amplification of the selection marker alone, possible in cases of expression vectors with the separate selection marker gene with its own promoter and terminator.

Human FSH was used as the target heterodimeric protein for testing the vector combination design because it is a practically useful and abundantly glycosylated protein. In this paper, we present the development of CHO cell line, secreting mostly the heterodimeric FSH in large quantities and the process of FSH purification. The resulting FSH biosimilar was tested at the comparative preclinical study [20] and at the clinical level to prove its bioequivalence at pharmacokinetics study [21] and at pharmacodynamics study in women undergoing in vitro fertilization (NCT03088137).

## Materials and methods

### Molecular cloning

Generation of the expression vectors p1.1 and p1.2-Hygro vectors is described previously [16]. PCR reagents and PCR Clean-Up System were from Evrogen, JSC (Moscow, Russia). Plasmids were isolated with a GeneJet Plasmid Purification Kit (Fermentas, Vilnius, Lithuania). The *Escherichia coli* TOP10 strain (Invitrogen, Carlsbad, CA) was used for cloning.

Sequences of FSH subunit ORFs were obtained from GenBank: NM_000510.2 (http://www.ncbi.nlm.nih.gov/nuccore/66528900)–for FSH β subunit, NM_000735.3 (http://www.ncbi.nlm.nih.gov/nuccore/NM_000735.3)—for FSH α subunit. The encephalomyocarditis virus (EMCV) internal ribosomal entry site (IRES), wtIRES («preferred-minimal» till 11 ATG), was from [19] corresponding to DQ288856.1 (149–713). The attIRES was from p1.1-F8BDD [17] (KY682701.1, 10855–11442). Two synthetic fragments with the different order of subunits ORFs as “α-subunit-IRES-β-subunit” (FSH-AIB) and “β-subunit-IRES-α-subunit’ (FSH-BIA) flanked with restriction endonucleases sites were synthesized by the Genscript, USA and cloned into the pUC57-Kan plasmid vector (GENEWIZ, Inc., USA) resulting in pUC57-Kan-FSH-AIB and pUC57-Kan-FSH-BIA plasmids, respectively.

Plasmids pUC57-Kan-FSH-AIB and pUC57-Kan-FSH-BIA were restricted by *Abs*I (Sibenzyme, Novosibirsk, Russia) and *Nhe*I (Fermentas, Vilnius, Lithuania) enzymes. The fragment encoding FSH subunits ORFs, IRES and the consensus Kozak sequence (GCCGCCATGG) [22] were transferred into p1.1 vector, restricted by the same enzymes resulting in the p1.1-FSH-AIB and p1.1-FSH-BIA plasmids respectively.

Plasmid pUC57-Kan-FSH-BIA was restricted by *Abs*I and *Spe*I (Sibenzyme, Novosibirsk, Russia). The fragment encoding FSH β subunit ORF with the consensus Kozak sequence was transferred into the p1.2-Hygro restricted by *Abs*I and *Nhe*I, resulting in the p1.2-Hygro-FSH-B-chain plasmid.

Expression plasmids for the transfection were purified by EndoFree Plasmid MaxiKit (Qiagen, Valencia, CA, USA) and sequence-verified (primers are listed in the S1 Table).

For the stable cell line generation, expression plasmids were linearized by enzymes cutting inside ampicillin resistance gene *bla* sequence. p1.1-FSH-AIB and p1.1-FSH-BIA plasmids were cut by the *Pvu*I (Fermentas, Vilnius, Lithuania) enzyme, p1.2-Hygro-FSH-B-chain was linearized by *Cci*I (Sibenzyme, Novosibirsk, Russia).

### Cell culture

A DHFR-negative CHO DG-44 cell line (cat # A1097101, ThermoFisher Scientific, Waltham, MA, USA) was cultured in the shake flasks in the chemically defined suspension medium CD DG-44, supplemented with 0.18% Pluronic F-68 and 4 mM L-glutamine (ThermoFisher Scientific). The culture flasks were maintained in a humidified incubator, 37°C/ 8% CO2, on a shaker, at a constant rotation rate of 130 rpm. DHFR-positive CHO S cell line (cat # R80007, ThermoFisher Scientific) was cultured in the shake flasks in the defined serum-free suspension medium, ProCHO 5 (Lonza, Basel, Switzerland), supplemented with 0.1% Anti-Clumping Agent (ThermoFisher Scientific) and 8 mM L-glutamine (ThermoFisher Scientific). The culture flasks were maintained in a humidified incubator, 37°C/ 5% CO2, on a shaker, at a constant rotation rate of 130 rpm.

The cells were passaged 24 h before transfection. Plasmids were transfected by electroporation in Gene Pulser Electroporation Buffer (Bio-Rad, Hercules, CA, USA) using a cuvette with a 4 mm gap with 7.5 million cells and 15 μg of linearized DNA for each transfection. Cells were counted by trypan blue exclusion and fluorescence microscopy at 48 h post-transfection.

Optimal conditions for a stable transfection were determined by dividing the transiently transfected populations into three parts, transferring them into culture medium, supplemented with 0.2; 0.5; 1 μM MTX for DG-44 cell line and 0.5; 1 or 2 μM MTX for CHO S cell line for 22 days with medium exchange every 3 days until the cell viability increased to 85%. Transfection efficiency was determined using the control plasmid pEGFP-N2 (Takara Bio USA / Clontech, Mountain View, CA, USA) and was about 6% in both cases.

The target gene amplification was performed for stably transfected cell population by stepwise increases of the MTX concentration until cells were not able to restore 85% viability in 15 days of culturing.

The selected MTX-amplified cell population was transfected with p1.2-Hygro-FSH-B-chain plasmid by electroporation as described previously. Hygromycin B (ThermoFisher Scientific) was added up to 750 μg/ml after 48 h. MTX concentration was maintained at 8 μM throughout all selection period. After 24 days of cultivation cell viability returned to 82% and antibiotic concentration was decreased to the less toxic maintenance level (250 μg/ml).

Clonal cell lines were generated from the final polyclonal population by limiting dilutions method. Cells were cultured in the shake flasks for 6 days without MTX and hygromycin and passaged 48 and 24 hours before seeding into 96-well plates. Cells were diluted up to 0.5 cell/well with EXCELL-CHO (Sigma) culture medium supplemented with 8 mM glutamine, 2 mM hypoxanthine and 2 mM thymidine (all ThermoFisher Scientific) and seeded into 96-well plates (200 μl/well). Plates were incubated 20 days at 37°C, 5% CO2 atmosphere prior to inspection. Wells with single colonies were marked and plates were incubated for 4 more days.

FSH concentration in culture medium was determined by ELISA and colonies with high expression level were subsequently transferred in 48-, 24- and 6-well plates. Six colonies were successfully re-adapted to suspension culture in the ProCHO 5 medium without MTX, hygromycin, hypoxanthine and thymidine; their doubling time, viability and FSH titer dynamics were determined.

### FSH purification

CHO cells were expanded in the shake flasks and seeded at 0.2 mln cells/ml in the four 0.5 L shake flasks with 125 ml ProCHO 5 culture medium each supplemented by 4 mM glutamine and 0.1% Anti-Clumping Agent and cultivated without medium change for 10 days. Cell mass was removed by centrifugation (400 g for 10 min in the fixed-angle rotor), and the conditioned medium was treated at room temperature by 0.3% TNBP and 1% Tween 80 for 6 hours for viral inactivation. After this pH of the medium was adjusted to 5.5 by the HCl and the treated medium was clarified by additional centrifugation (14000 g, 20 min in the in the fixed-angle rotor).

The first step of chromatographic purification was carried out on the 10 ml Tricorn GL 10/100 column with 10 ml of the CaptoMMC resin (both GE Healthcare Bio-Sciences, Pittsburgh, PA, USA), equilibrated with 50 mM Na-phosphate pH 5.5, 100 mM NaCl solution. The clarified medium was pumped through the column at 5 ml/min, after this the column was washed by 100 ml of the equilibration solution. FSH was eluted by the 50 mM Tris-HCl pH 7.5, 150 mM NaCl solution and applied undiluted at 2.5 ml/min to the 10 ml column with the CaptureSelect FSH-Affinity Matrix (ThermoFisher Scientific) resin, equilibrated by the 20 mM Tris-HCl pH 7.5, 150 mM NaCl solution. The column was washed by 100 ml of the equilibration buffer and by 50 ml of the 20 mM Tris-HCl pH 7.5, 500 mM NaCl solution. FSH was eluted from the affinity column by the 50 mM Tris-HCl pH 7.5, 2 M MgCl_2_ solution. Approximately 25 ml eluate was collected. The solution of the purified FSH was desalted in 10 ml portions by the XK 26/10 column with the 80 ml Sephadex G-25 fine (both GE Healthcare Bio-Sciences) resin. The column was equilibrated by the 10 mM Na-phosphate solution, pH 7.0. Desalted FSH solution was subjected to the tandem chromatography—passed through the Sartobind S membrane (Sartorius Stedim Biotech, Goettingen, Germany), attached to the 1 ml Capto Q column (GE Healthcare Bio-Sciences) at 1 ml/min. The assembly was equilibrated by the same solution and washed by 10 ml of the equilibration solution after application of the FSH solution. The Sartobind S membrane with bound admixtures and leaked single-domain antibodies were removed and the Capto Q column was re-equilibrated by the 20 mM Tris-HCl pH 8.5 until the pH of the eluate was 8.5. Excess neutral isoforms of the FSH were removed by the 5 ml of the 20 mM Tris-HCl pH 8.5, 60 mM NaCl solution, more acidic FSH isoforms were eluted by the 20 mM Tris-HCl pH 8.5, 200 mM NaCl solution, utilizing reverse flow direction at 0.5 ml/min.

The oligomers and the free FSH chains were separated from the heterodimeric FSH molecules by the size exclusion chromatography, utilizing Tricorn 60/100 column with the Superdex 75 resin (GE Healthcare Bio-Sciences). Column was pre-equilibrated by the 10 mM Na-phosphate pH 7.0 solution at the 0.5 ml/min flow. FSH solution was applied by 2 ml portions, the main peak of the heterodimeric FSH was collected; collection of the eluate was stopped at 10% of the peak’s height in order to remove the free chains, eluting as the main peak’s right shoulder. Pure FSH in the Na-phosphate solution was passed through the viral filtration membrane Virosart CPV (Sartorius Stedim Biotech), supplemented by L-methionine up to 0.5 g/L, sucrose up to 68.46 g/L, Tween-20 up to 0.02 g/L, sterile filtered and stored frozen in aliquots at -70 °C for further use.

### Glycan separation and analysis

The glycan profile of the purified FSH was analyzed and quantified using normal-phase (NP) and weak anion exchange (WAX) HPLC column. The analysis was done in combination with enzymatic degradation of the glycans. For NANA and NGNA analysis, sialic acid was enzymatically cleaved from FSH. The sialic acid was labeled with DMB (fluorescence dye that specifically labeled sialic acid residues), separated by RP-UPLC column and quantified using sialic acid standards mixtures.

FSH sample was desalted by the desalting column prior to enzymatic glycan release by the PNGase F. Cleavage was verified by SDS-PAGE. Glycans were separated from other impurities and labeled with 2-aminobenzamide (2AB). 2AB-labeled glycans were separated on the normal phase (NP) column and weak anion exchange (WAX) column using HPLC. Additional enzymatic degradation of glycans was performed using sialidase, β1,4 galactosidase, and β1,4 galactosyltransferase. Glycans were identified according to retention time database built using in-house-prepared standards. Most peaks were verified by enzymatic degradation of the glycans followed by re-separation on the NP column.

Sialic acids were cleaved from the desalted FSH sample by the sialidase treatment, triplicates were analyzed. Sialic acids were labeled with DMB (1,2-diamino-4,5-methylenedioxybenzene dihydrochloride) and separated by the HPLC reverse phase (RP) column. Identification of sialic acid types was performed according to their retention time using pure sialic acid standards. Quantification of sialic acids was done using the calibration curve, generated for the DMB-NANA (N- acetylneuraminic acid).

### FSH-binding assay

FSH receptor expressing cells (cat # HTS178RTA, Eurofins Discovery Services, Dundee, UK) were used for kinetic study. Cells were grown in DMEM (90%), Fetal bovine serum (10%), 100 U/ml penicillin and 100ug/ml streptomycin in T-175 vented flasks at 37°C, 5% CO_2_, in culture media changed every 2–3 days. For membrane preparation cells were homogenized in the lysis buffer—50mM Tris-HCl, 5 mM MgCl_2_, 5 mM EDTA, protease inhibitor cocktail (all Sigma). The homogenate was centrifuged at 14,000 x g for 10 minutes at 4°C to pellet the membranes. The pellet was resuspended in fresh buffer and re-centrifuged. The pellet was then resuspended into the same buffer (5 ml) containing additional 10% glucose as a cryoprotectant, divided into 0.5 ml aliquots and stored at -80°C.

Radioligation of FSH was performed as follows: 10 mM Na-phosphate pH 7.0 (60 μL), FSH protein solution (14 μg), lactoperoxidase (2 μg) (Sigma), Na^125^I (0.25–0.5 mCi), and H_2_O_2_ up to 4 mg/L were added to an Eppendorf vial. After 30 min the reaction was quenched with sodium bisulphite and ascorbic acid. The radioiodinated FSH was separated from unreacted radioiodine using a G-25 desalting column (GE Healthcare). The labeled FSH was formulated with 1% BSA and 10% sucrose, divided into aliquots and stored at -20°C. Radiochemical yield was > 90%.

The radioligand binding assay was carried out in a final volume of 250 μL per well. The radiolabeled FSH specific activity was adjusted with unlabeled FSH to give the final FSH concentration for the assay (0.65 nM). To each well 200 μL buffer was added followed by 50 μL of radiolabeled FSH solution in the same buffer at various time points prior to the termination of the assay (shortest—30 seconds to 1 minute; longest 180 minutes). Radiolabeled FSH additions were spaced by 15 sec between compounds to allow time for addition and mixing. For non-specific wells, 150 μL buffer was added to each well followed by the 50 μL of unlabeled FSH solution and 50 μL of the radioligand solution in buffer. The incubation was stopped by vacuum filtration onto presoaked Whatman GF/C filters (Sigma) using a 96-well FilterMate harvester (Perkin Elmer, Waltham, MA, USA) followed by five washes with ice-cold wash buffer. Filters were dried under a warm air stream, sealed in the polyethylene film, scintillation cocktail was added and the radioactivity counted in a Wallac TriLux 1450 MicroBeta (Perkin Elmer) counter. For each concentration or time point non-specific binding was subtracted from total binding to give specific binding. Data were fit using the non-linear curve fitting routines in Prism (Graphpad Software Inc, San Diego, CA, USA). Dissociation curves were fit using a one-phase model. The final plateau level was set to zero and the initial (time zero) value obtained following either buffer or buffer with unlabeled ligand addition immediately prior to filtration. Association curves were fit using a single concentration of FSH and using K_off_ values from the dissociation experiments.

### ELISA

ELISA was performed with FSH ELISA kit (DRG Instruments Gmbh, Marburg, Germany) according to user manual. Test samples were diluted 1000x to 40000x by the Tris-based saline with 1% BSA, test samples dilutions, producing the primary readings in the 0.25–1 AU range were used for calculations. Calibration curve was generated by the linear regression method.

### Quantitative PCR, PCR, RT-PCR

The transgene copy number in the CHO genome was determined by the quantitative real-time-PCR (qPCR) as described in [18]. Serial dilutions of highly purified p1.1-AIB or p1.2-Hyg-eGFP [16,17] plasmids were used for calibration curves. PCR results were normalized to data obtained for primers to peptidyl-prolyl isomerase B (*PPIB*) gene region, presumably unique to the CHO cells genome according to the BLAST search results. Weight of one CHO haploid genome was taken as 3 pg according to [23]. Primers are listed in the S1 Table, qPCRmix-HS SYBR reaction mixture (Evrogen) and iCycler iQ thermocycler (Bio-Rad) were used. Calculations of threshold cycles, calibration curves, PCR efficiency, and copy numbers were made by the iCycler Iq4 program. All determinations were repeated 3 times, in 3–5 replicates, sample volume 25 μl.

### Western blotting

Samples of the conditioned medium were clarified by centrifugation and subjected to 12% SDS-PAGE in non-reducing conditions. Protein transfer, blocking, hybridization and color development were done according to [24] using Hybond C Extra membrane (GE Healthcare) and a 3,3’,5,5’–tetramethylbenzidine substrate (Sigma). Protein transfer was done by the electroblotting, utilizing the TE 70 PWR apparatus (GE Lifesciences) and the Towbin solution. Membrane blocking was performed in the 20 mM Tris-HCl, pH 7.6, 150 mM NaCl, 5% bovine serum albumin (BSA, Sigma) 0.05% Tween-20 solution, membrane wash was performed by the 20 mM Tris-HCl, pH 7.6, 150 mM NaCl, 0.05% Tween-20 solution, hybridization—in the 20 mM Tris-HCl, pH 7.6, 150 mM NaCl, 0.5% BSA solution. Mouse monoclonal antibody toward gonadotrophic hormones common α-subunit, HRP conjugate, was purchased from Hema-Medica, Moscow, Russia (cat # К203) and used undiluted, mouse monoclonal antibody toward FSH β-chain was used in the 1:2000 dilution (cat # ab47161, Abcam, Cambridge, UK) and anti-mouse IgG goat antibody HRP conjugate was used at 1:2000 dilution (Sigma). Hybridization with all antibodies was done at room temperature for 1 hour.

### Isoelectrofocusing (IEF)

Fifty μg FSH samples were desalted by acetone precipitation and dissolved in IPG-buffer, pH 4–7 (GE Healthcare). IEF was performed in Ettan IPGphor3 (GE Healthcare) under non-denaturing conditions with Immobiline DryStrip pH 3–10 NL gels (GE Healthcare). Gel strips were fixed by 30% ethanol and 12,5% trichloroacetic acid and stained by colloidal Coomassie solution (ThermoFisher Scientific).

### Fluorescence *in situ* hybridization (FISH)

Colchicine (Sigma) in a concentration of 0.05 μg/ml was added in the culture flasks and the cells were incubated for 4–6 h after which the cells were exposed to the hypotonic solution (0.075 M KCl) and fixed in methanol:glacial acetic acid (3:1) mixture. The cytogenetic FISH analysis was carried out using locus-specific DNA probes:

5’-FITC-CCAAAGGAATGCAAGGTCTGTTGAATGTCGTGAAGGAAGC

5’-FITC-TAAGGCCGGTGTGCGTTTGTCTATATGTTATTTTCCACCATATTGCCGTC.

The hybridization mixture with DNA probes was denaturated at 75°C for 5 minutes and incubated at 37°C for 30 min. The cytogenetic preparations were denaturated with 70% formamide at 75°C for 5 min and hybridized at 37°C overnight. On the next day, the preparations were washed with 0.4×SSC solution (72°C) 2 min on a water bath, after that with 2xSSC-Tween (RT) 2 min. The metaphases were contrasted with 4',6-diamidino-2-phenylindole (DAPI). Metaphase analysis was carried out using a Metafer system and Metasystems ISIS software (Carl Zeiss AG, Feldbach, Switzerland).

### *In vitro* FSH activity assay

*In vitro* activity of purified FSH was analyzed by cell-based assay described earlier [25]. Subsequently, 4-parameter logistic dose-response curves were fitted to the background subtracted data sets on the basis of 9 serial 1:3 dilution steps with each concentration assayed in quadruplicates using PLA 2.0.0 software (Stegmann Systems, Germany). FSH samples were analyzed against a plate specific 2^nd^ international WHO standard for recombinant FSH (NIBSC 92/642).

### *In vivo* FSH activity assay

*In vivo* FSH biological activity was determined according to recommendations given in the European Pharmacopoeia, Ed.8.5 (Ch. 2285 and 2286) as described earlier [20], 21 rats per each group. This study was carried out in strict accordance with the GOST 33216–2014 “Guidelines for accommodation and care of animals. Species-specific provisions for laboratory rodents and rabbits”. The protocol was approved by the Institutional Commission for the Control of the Maintenance and Use of Laboratory Animals of the Institute of Bioorganic Chemistry, Russian Academy of Sciences (Protocol Number: 142/2014). All animals were euthanized in the CO_2_ chamber before dissection, and all efforts were made to minimize suffering.

## Results and discussion

### Construction of expression plasmids

Recently developed vectors for stable high-level expression were used for a novel FSH-producing cell line generation. The p1.1 vector and its derivative p1.2-Hygro [16, 17] contain strong constitutive promoters and elements for improving genome integration and amplification of the transgene. The simultaneous expression of two target genes was provided by the encephalomyocarditis virus IRES element (EMCV) allowing 5’cap-independent translation initiation.

**Fig 1:**
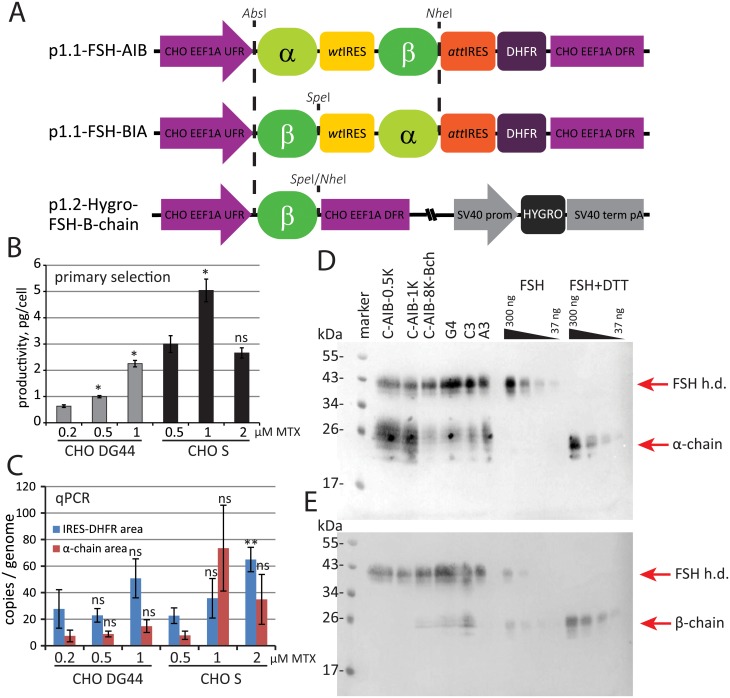
Scheme of the genetic constructions and the characterization of the primary stably transformed cell populations. A—Scheme of the tricistronic (p1.1-FSH-AIB, p1.1-FSH-BIA) and bicistronic (p1.2-Hygro-FSH-B-chain) expression plasmids. CHO EEF1A1 UFR—upstream flanking area of the EEF1A1 gene (elongation factor-1 α gene promoter, flanked with 5’ UTR), DFR—downstream flanking area (elongation factor-1 α gene terminator and polyadenylation signal, flanked with 3’ UTR), EMCV IRES—encephalomyocarditis virus internal ribosome entry site (wild type); attEMCVIRES—attenuated encephalomyocarditis virus internal ribosome entry site; DHFR—ORF of the dihydrofolate reductase gene, a—ORF of the FSH α-subunit; b—ORF of the FSH β-subunit, SV40 promoter—promoter of the polyomavirus simian virus 40; SV40term, pA—terminator and polyadenylation signal of the polyomavirus simian virus 40, hygroB—Hygromycin B resistance gene (hygromycin B phosphotransferase). Restriction sites used for cloning are shown in italics. B—FSH secretion level in the stably transformed cell populations by ELISA. Data are mean, n = 2, *—P-value <0.05, **—P-value <0.01, ns—P-value >0.05 by the t-test. C—qPCR analysis of the expression cassette copy numbers per haploid genome. IRES-DHFR area—RT-ID-F, RT-ID-R primer pair; a-chain area—SQ-FA-F, SQ-FA-R primer pair. All data normalized to the PPIB copy number. Error bars represent standard deviation, n = 3. D—Western blotting of secreted FSH in culture medium, antibodies to α-chain. C-AIB-0.5K—stably transfected CHO S cell line 0.5 μM MTX, C-AIB-1K—polyclonal population obtained at 1 μM MTX, C-AIB-8K-Bch—polyclonal population amplified up to 8 mkM MTX and additionally transfected by the p1.2-Hygro-FSH-B-chain plasmid; G4- monoclonal cell line C-P1.3-FSH-G4, C3—monoclonal cell line C-P1.3-FSH-C3, A3—monoclonal cell line C-P1.3-FSH-A3; FSH—Gonal F, FSH+DTT—Gonal F pretreated with 10mM DTT, “FSH h.d.”–FSH heterodimer. SDS-PAGE in non-reducing conditions, molecular weights are shown in kDa. E—Western blotting of secreted FSH in culture medium, antibodies to β-chain. Same samples to panel D, another membrane.

Relative expression levels for two ORFs of FSH subunits interspersed by the wild type (wt) EMCV IRES are not currently known, so we have constructed two expression plasmids, containing sequences α-chain-IRES-α-chain (AIB) and β-chain-IRES-α-chain (BIA). Since it was not possible to create by restriction-ligation steps the DNA fragment containing the wtEMCV IRES and the FSH chain ORF starting exactly at the start codon 12, the AIB and BIA fragments were constructed from synthetic oligonucleotides (Genescript, Piscataway, NJ, USA). Codon optimization for FSH chains ORFs gave contradictory results earlier [11], the reported expression level from the synthetic ORFs did not exceed that of the full-length natural sequences [12,26], so we used the natural sequences of both ORFs. Two synthetic sequences AIB and BIA were cloned into the p1.1 vector, resulting in the p1.1-FSH-AIB and p1.1-FSH-BIA plasmids (Fig 1A). The ORF areas of the FSH α-chain and β-chain were extracted from cloned AIB and BIA fragments by restriction and sub-cloned to the p1.2-Hygro vector, resulting in p1.2-Hygro-FSH-A-chain and p1.2-Hygro-FSH-B-chain plasmids, respectively (Fig 1A). These plasmids have a Hygromycin B resistance gene instead of the DHFR selection marker. They can be stably transfected to the cells, which are already transfected by the p1.1-derived plasmids with the DHFR marker.

### Generation of stably transfected cell populations by the p1.1-based plasmids

The p1.1-FSH-AIB and p1.1-FSH-BIA plasmids were linearized in the area of the ampicillin resistance gene and transfected to the DHFR-deficient CHO DG-44 cells and to the DHFR-positive CHO-S cells. For both plasmids, CHO-S cells showed the significantly higher level of the FSH expression 48 h post-transfection at the nearly the same transfection efficiency—35 ng/ml in the case of p1.1-FSH-AIB plasmid and CHO DG44 cells and 130 ng/ml in the case of CHO-S cells. Although the reason for this large difference is not clear, it is consistent with the difference in productivity between stably transfected cell populations (see below). Stably transfected cell populations were generated in the presence of various concentrations of DHFR inhibitor MTX. In the case of p1.1-FSH-BIA plasmid, both cell lines secreted no detectable levels of FSH at all MTX levels, and in the case of p1.1-FSH-AIB plasmid, all six established cell populations secreted significant quantities of FSH (Fig 1B).

Copy numbers of genome-integrated plasmids were determined in stably transfected populations by qPCR (Fig 1C). In the case of CHO-S host cells, copy numbers for both amplicons are similar, but in the case of CHO DG44 host cells copy numbers for two different amplicons are imbalanced, probably due to partial loss of some plasmid areas upon integration. Additionally, it was found that CHO-S cells stably transfected by the p1.1-FSH-BIA plasmid have multiple copies of genome-integrated DHFR, but the β-chain ORF was not present at all (S1 Fig). We assumed that plasmid p1.1-FSH-BIA was fully cleaved in the area of β-chain ORF upon integration into cell’s genome, resulting in complete loss of heterodimeric FSH secretion.

Surprisingly, stably transfected DHFR-positive CHO-S cells also showed higher productivity than the DHFR-negative DG-44 cells. Most productive cell population, derived from CHO-S cells and obtained in the presence of 1 μM MTX (cell population code C-AIB-1K), was used for further MTX-driven target gene amplification. Expectedly, free α-chain, but not the free β-chain, was secreted to the culture broth together with the desired FSH heterodimer by the cells, stably transfected by the p1.1-FSH-AIB plasmid, according to the Western blot data (Fig 1D and 1E).

**Fig 2:**
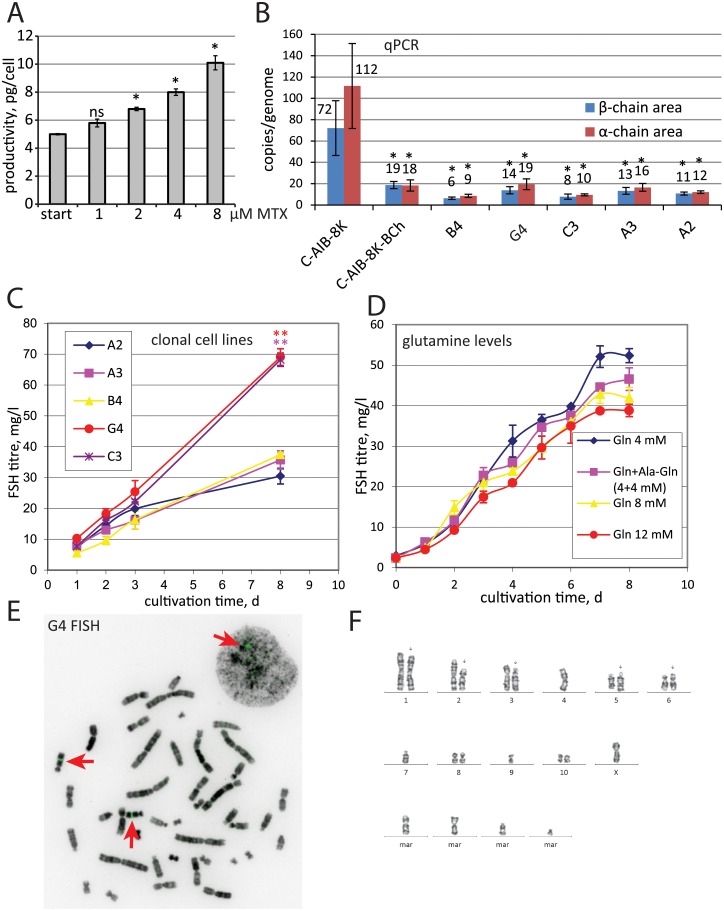
Productivity and transgene copy number for oligoclonal cell populations during the MTX amplification and for selected clonal cell lines. A—–Secretion level of FSH for cell populations upon MTX amplification by ELISA, n = 2. B—qPCR analysis of the expression cassette copy numbers per haploid genome for the oligoclonal population after the amplification, second transfection and subsequent cell cloning. B-chain area—primer pair SQ-FB-F—SQ-FB-R. Error bars represent standard deviation, n = 3. C—FSH accumulation curves by ELISA for selected clonal cell lines during batch cultivations, n = 2, t-test analysis of G4 and C3 samples versus A2 sample, only statistically significant differences are marked by asterisks. D—Effects of glutamine concentration on the FSH secretion level for the G4 cell line. E—FISH analysis of the G4 cells by the FITC-labeled probes toward the α-chain area (green channel). Arrows depict positions of fluorescent bands at condensed chromosomes and the interphase nuclei. F—Karyotype of G4 cells. Arrow indicates the probe location (at long-arm, chromosome 6).

Three consecutive steps of cell cultivation in presence of increasing MTX concentrations were performed for the C-AIB-1K cell population, 15 days at each MTX level. Resulting cell population (code C-AIB-8K) has approximately two times higher productivity than the initial one (Fig 2A).

### Generation of polyclonal cell population stably transfected by p1.1-FSH-AIB and p1.2-Hygro-FSH-B-chain plasmids

Immunoblotting assay with monoclonal antibodies against the FSH subunits demonstrated the significant predominance of the free α-subunit in culture medium derived from C-AIB-8K cells cultivation (Fig 1D). Both control and test samples of the FSH were significantly retarded on the gel, probably due to high content of the oligosaccharides, but demonstrated similar electrophoretic mobility. Alpha subunit was distributed between heterodimer and monomer states and β-subunit was found only in heterodimer FSH that could be explained by the sub-optimal translation re-initiation of β-subunit ORF from EMCV IRES.

The C-AIB-8K cells were transfected with 25% efficiency by the p1.2-Hygro-FSH-B-chain plasmid coding the FSH β-subunit and the hygromycin resistance marker. No changes in FSH expression level were seen 48 h post-transfection but after the generation of stably transfected cell population in the presence of 750 μg/ml of selection antibiotic hygromycin B specific FSH titer increased twice and was 23.5 pg/cell, that was at least ten times higher, that of previously published FSH-producing cell lines. The resulting polyclonal cell population C-AIB-8K-Bch was able to secrete FSH up to 81 μg/ml at 12 days of cultivation without medium changes, specific productivity was 4.7 pg/cell/d. RT-PCR data demonstrated significant drop of both chain’s ORF areas copy numbers, that could be a consequence of inactive copies deletion or reduction of cells subpopulation bearing the highest number of integrated genes upon the second transfection and selection process (Fig 2B).

### Generation of monoclonal FSH-producing cell lines

Clonal FSH-producing cell lines were derived from the C-AIB-8K-Bch population by limiting dilutions. We obtained 330 single colonies and selected six rapidly dividing cell clones with highest FSH titers. After re-adaptation of these clones to suspension culturing FSH concentration in their culture medium were determined (Fig 2C). Specific productivity of monoclonal cell line C-P1.3-FSH-G4 (below will be denoted as G4) was 12.3±1.7 pg/cell/day. Two backup cell lines C3 and B4 also demonstrated comparable specific productivities– 9.6±0,1 pg/cell/d and 7.4±0.7 pg/cell/d, respectively. This high specific productivity level was in line with the immunoblotting data, showing only marginal level of the free α chain for the G4 cell line and higher proportion of the free α chain for other clonal lines and the predominance of the free α chain of the FSH in the parent cell population (Fig 1D). This distribution of α-chains between heterodimeric FSH molecules and the free state was also in agreement with the copy number data (Fig 2B).

### The optimization of the cultivation process of monoclonal FSH-producing cell line C-P1.3-FSH-G4

In the first round of optimization procedure, C-P1.3-FSH-G4 cells were cultivated in ProCHO 5 (Lonza) culture medium without selection agents. The effects of different supplements as Anti-Clumping Agent (Gibco) and GlutaMAX-I Supplement (Gibco) were examined. Anti-Clumping Agent could increase cells viability in high-density cultures and GlutaMAX could serve as the prolonged and stable source of glutamine as compared to the free amino acid. Neither Anti-Clumping Agent treatment nor equimolar substitution of Gln for Glutamax had any effect after 4 days of cultivation (data not shown). At the same time, the level of FSH was dependent on the glutamine concentration, as 12 μM of glutamine diminished the product titer compared to 4–8 μMμ (Fig 2D).

Ready-to-use liquid ProCHO 5 medium was compared to the same medium, obtained as the powder and prepared on site as well as to another protein-free medium concentrate ChoMaster HP-5 (Cell Culture Technologies LLC, Ticino, Switzerland), diluted by the sterile water in-house and supplemented by 1 and 10 mg/L insulin. It was observed that the FSH titer was steadily increasing for 5 days of culture in each case (S2 Fig), but only the ProCHO 5 medium allowed it to increase for 8 days of batch culture, probably due to the higher content of glucose and amino acids from the natural source. Therefore, we considered ProCHO 5 medium supplemented with Anti-Clumping Agent and 4 mM glutamine to be the optimal medium for culturing FSH-producing cells.

### FSH expression level stability in long-term periodic culturing

**Fig 3:**
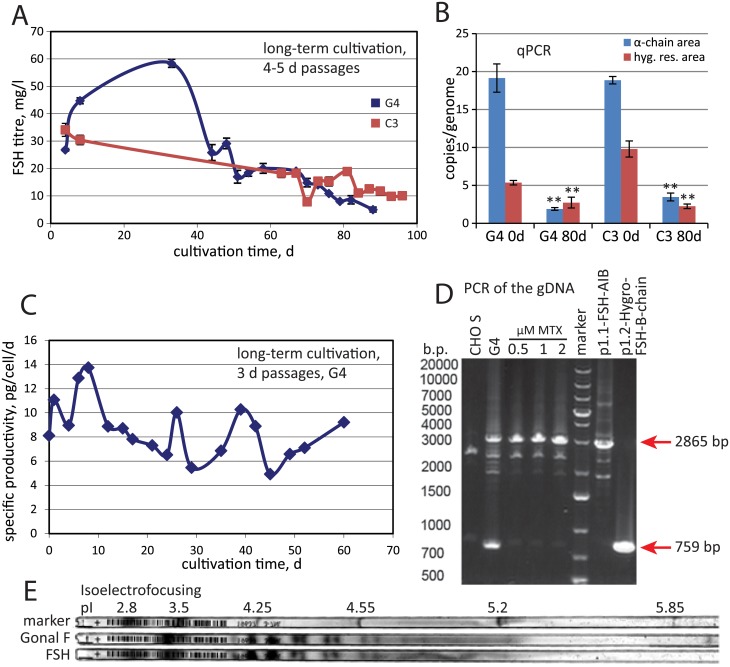
The long-term secretion rate dynamics, genetic stability of the selected clonal cell line and hormone isoforms distribution of the purified FSH. A—FSH titre dynamics for the long-term cultivation of the G4 and C3 clonal cell lines, 4–5 days passages by ELISA, n = 2. B—qPCR analysis of the expression cassette copy numbers per haploid genome for clonal lines after long-term cultivation. “hyg. res. area”—Hygromycin B resistance gene area, primer pair RT-HYG-F—RT-HYG-R. Error bars represent standard deviation, n = 3. 0d –the first day of cultivation, 80d –samples taken after the 80 days of cultivation. C—Dynamics of the specific productivity of the G4 cell line during long-term cultivation, 3 days passages. D—PCR analysis of the G4 cell line genomic DNA, primers pair SQ-5CH6-F—SQ-3CH1-R. Expected products sizes are depicted by arrows, the image was contrast-enhanced to add visibility. E—Isoelectrofocusing analysis of the purified FSH. Samples were loaded in non-denaturing conditions.

Two clonal cell lines with the highest specific productivity, G4 and C3 were tested for long-term expression level stability in the non-selection medium without MTX, hygromycin, hypoxanthine and thymidine. Initially, both cell lines were passaged to the 0.25 mln cells/ml seeding culture density and cultivated for 4–5 days in the shake flasks before the next passage. Maximally allowed cell density at the moment of passage was set as 4 mln cells/ml. Both cell lines demonstrated significant product titer drop at 64 days of culturing (Fig 3A), accompanied by the loss of most genome-integrated genetic cassettes (Fig 3B). Cultivation was performed in the absence of MTX and hygromycin to mimic the conditions for large-scale industrial culturing.

We noticed that the growth rate of cells after 64 days of culturing was significantly less than in the beginning. For the G4 cell line, growth rate changed from 1.27 d^-1^ to 1.46 d^-1^ and for the C3 line—from 0.99 d^-1^ to 1.7 d^-1^. We reasoned that the observed drop in productivity could be attributed to the long intervals between passages and high cell density, forcing the outgrowth of the genetically altered cells, dividing more rapidly and producing lesser amounts of the product. To test this, we lowered the density and shortened the passage time, for the most productive cell line G4, using the seed density 0.2 mln cells/ml, three days planned passage time and maximal allowable cell density 1.8 mln cells/ml. Indeed, no significant change of the product titer was observed during 60 days of culturing (Fig 3C) under these conditions in the absence of selection agents, thus demonstrating sufficient stability for further cell bank generation and the industrial use. Cell line G4 was additionally tested for genetic insert consistency by PCR analysis of the extracted genomic DNA at the moment of master cell bank generation, 12 d of passaging from the initial research cell bank (Fig 3D). It was found that target genes areas in both genome-integrated plasmids gave PCR products of expected sizes, consistent with the absence of large deletions or insertions in the target protein genes areas. Absence of mutations in both ORFs was also verified by PCR amplification of corresponding ORF areas, molecular cloning of PCR products into the plasmids and sequencing of the inserts from 3x2 plasmid clones. No mutations were detected (data not shown) in both FSH chain’s ORFs.

### FISH and karyotype analysis

Fluorescence in situ hybridization (FISH) was used to confirm genomic integration of the plasmid. Metaphasic chromosomes and interphase nuclei of G4 cells were hybridized with fluorescently labeled DNA probes as described in Materials and methods. Hybridization of probes was located on q-arm of chromosome 6 (Fig 2E) and cytogenetic analysis revealed the major modal karyotype of G4 cell line as 22,-X,del(1)(p2.10),del(2)(p1.7p2.9),inv(3)(p2.7q1.3),add(5)(q3.2;?),add(6)(?;p1.1),-7,-9,+4mar (Fig 2F). No significant changes from the karyotype of CHO cells are visible.

### Purification of FSH

For the preparation of laboratory-scale purified FSH batches, G4 cells were cultivated in the shake flasks without medium change for 10 days. Cell mass was removed by centrifugation and the harvested medium was used for virus inactivation by the solvent-detergent treatment, utilizing Tween 80 and TNBP. Total FSH was purified first by the Capto MMC column, followed by the Capture Select FSH-Affinity Matrix column. Purified FSH was desalted on the Sephadex-G25 column, residual admixtures were removed by the Sartobind S membrane and the FSH was adsorbed to the Capto Q resin. Excess of neutral isoforms was removed by the 60 mM NaCl, 20 mM Tris-HCl solution wash, target FSH isoforms pool was eluted by higher NaCl concentration. Residual oligomers of the FSH and the free chains were separated by the SEC chromatography, at the same time FSH was transferred to the sodium phosphate solution. Fully purified FSH was passed through the viral filtration membrane, formulated by excipients, sterile filtered and stored in aliquots at -70 °C. This purification process involves only linearly scalable chromatography operations, so it can be employed at the industrial scale with virtually no changes.

### Analysis of the purified FSH

Purified FSH was tested by the isoelectrofocusing (IEF), using the follitropin alpha (Gonal-F, Merck Serono, Italy) as the band pattern standard (Fig 3E). FSH preparation, obtained from the G4 cell line by the simple batch culturing and purified as described above, showed a very similar band pattern to the follitropin alpha originator product on the IEF gel and ranged between pI 3–6. More detailed testing of N-linked glycans in the purified FSH is summarized in Table 1. Sialic acid in the purified FSH was presented mostly in the form of NANA and bi-acetylated NANA (9,4%), level of “non-human” sialic acid variant—NGNA was below 1% from total (S3 Fig), as we have reported earlier [17]. Most of the N-linked glycans according to the normal phase HPLC data belonged to the complex type with only minor content of high-mannose and hybrid types (S4–S7 Figs). Core fucosylation of the glycans was found to be moderate and highly branched structures are more fucosylated than the biantennary ones. This distribution of N-glycans structures, composition, and charges (S8 and S9 Figs) is comparable to the previously published data for CHO-derived recombinant FSH [20,27,28].

**Table 1:** Glycan structures and abundance in the purified FSH sample, obtained from the G4 cell line.

Structure name	Content, %
High mannose	2.6
Hybrid	1.2
Complex	96.2
mono-antennary- fuc /afuc^a^	n.d
bi-antennary- fuc /afuc	13.9/35.0
tri- antennary-fuc/afuc	14.2/11.6
tetra-antennary-fuc/afuc	11.1/5.1
**Charged glycans**	
Neutral	2.7
-1	21.2
-2	45.3
-3	21.2
-4	9.7
**Charge other than sialic acid**	
-1	2.2
-2	3.5

^a^ Fuc = with core fucose, afuc = without core fucose.

Interaction of the FSH with its receptor was studied by the radiolabeled ligand method. Association rate constant for the FSH was determined by the incubation of the membrane fraction, derived from the HEK293 cells, transfected by the human FSH receptor gene, with I125-labeled FSH preparations. Both association and dissociation curves (S10 Fig), generated for the control FSH sample (Gonal F) and two batches of the FSH, produced by the G4 cells and purified as described above, are similar, generating very similar Kon, Koff and KD constants (Table 2).

**Table 2:** Association and dissociation constants for the interaction of purified FSH with FSH receptor.

Sample	k_on_, nM^-1^*min^-1^	k_off_, min^-1^	t_1/2_, min	K_D_, pM
Gonal-F	0.89±0.23	0.0057±0.0017	134±40	6.3±2.5
FSH #1	0.71±0.13	0.0034±0.0006	209±35	4.8±1.2
FSH #2	0.65±0.16	0.0043±0.0008	167±31	6.6±2.1

Data are given as mean ± 95% confidence interval, n = 2.

### *In vitro* and *in vivo* FSH activity

Three samples of purified FSH were analyzed using cell-based assay. Specific activities of 10.5 IU/μg, 12.2 IU/μg, and 10.8 IU/μkg were determined (S11 Fig). This results in a mean specific activity of 11.2 IU/μg (+/- 8%) which corresponds to 88% of the specific activity of the international standard (Table 3). The measuring of *in vivo* activity of the purified FSH is based on analyzing the mass increase of rat ovaries in the dose-dependent concentration range. The biological activity of FSH was compared with WHO standard using linear regression (S12 Fig) in which the ovary mass was used as the response to the administered FSH (with PLA 2.0.0 software, Stegmann Systems). Thus, the determined *in vivo* activity of the purified FSH was 13.6 IU/μg (95% confidence interval: 12.3–15.1 IU/μg).

**Table 3:** Summary of derived specific *in vitro* activities for one FSH sample, slope ratios and upper asymptote ratios.

No (Replication)	Specific activity (IU/μg)	Specific activity % of standard	Slope ratio (sample/standard), %	Upper asymptote ratio (sample/standard), %
1	10.5	83	105	102
2	12.2	96	95	105
3	10.8	86	99	103

## Discussion

The aim of this study was to develop a new recombinant FSH producing cell lines with high specific productivity and sufficient long-term genetic stability. We used previously designed specialized plasmid vectors, containing non-coding areas of the EEF1A1 gene and the fragment of the long terminal repeat from the Epstein-Barr virus. Open reading frames of two FSH subunits were connected by the wild-type EMCV IRES and linked to the mouse DHFR ORF area by the attenuated EMCV IRES, forming the tricistronic mRNAs. It was found that secretion of heterodimeric FSH is evident only in the case of α-chain ORF in the first cistron position, the opposite order of chain’s ORFs resulted in non-secreting stably transfected cell population. DHFR-positive CHO S cells, stably transfected by the p1.1-FSH-BIA, demonstrated much higher specific productivity, than DHFR-negative CHO DG44 cells. Excess of the free α-chain of FSH in the culture medium was compensated by the second transfection of the polyclonal cell population by the plasmid, coding only the β-chain of FSH. Several of the clonal cell lines derived from the doubly transfected cells secrete heterodimeric FSH in large quantities. Therefore, the combination of genome-amplifiable tricistronic plasmid vector and monocistronic vector with the DHFR-compatible selection marker is sufficient for generating cell lines secreting large quantities of heterodimeric glycoprotein hormones.

Since the best-producing clonal cell line C-P1.3-FSH-G4 maintains the constant titer of secreted FSH for at least 2 months of cultivation in batch culture, it may be used for large-scale long-term FSH production. Purified FSH, derived from the C-P1.3-FSH-G4, was analyzed on receptor binding, glycan structure, FSH *in vitro* and *in vivo* activity. It has very similar structural properties to other original and biosimilar FSH preparations described in the literature. Taking together, we have shown that EEF1A1-based vectors with multicistronic operons are suitable for the generation of cell lines producing large quantities of heterodimeric proteins for therapeutic applications in humans. High specific cell productivity achieved using our approach may have significant implications for industrial applications–much smaller bioreactor vessels, simple batch culturing instead of perfusion-based process and complete absence of ultrafiltration-diafiltration operations will greatly diminish both capital and operational costs of biosimilar FSH production.

A method for clonal cell line generation resulting in large quantities of secreted heterodimeric proteins with only minor levels of free chain by-products, as described in this article, may be a promising general strategy for production of various glycosylated heterodimeric hormones, including lutheinizing hormone, thyroid-stimulating hormone, chorionic gonadotropin and other biotherapeutic proteins with the comparable size and level of glycosylation.

## Supporting information

S1 Fig qPCR analysis of the expression cassette copy numbers per haploid genome for p1.1-FSH-AIB and p1.1-FSH-BIA plasmids. IRES-DHFR area—RT-ID-F, RT-ID-R primer pair; β-chain area—SQ-FB-F, SQ-FB-R primer pair. All data normalized to the PPIB copy number. Error bars represent standard deviation, n = 3.
(EPS)

S2 Fig Effects of culture media variants on the FSH secretion level of the selected clonal cell lineG4. 1 –liquid ProCHO 5 medium, 2 –powdered ProCHO medium, 3 –diluted CHO Master medium concentrate with 1 mg/l insulin, 4 –CHO Master with 10 mg/l insulin. FSH concentrations measured by ELISA.
(EPS)

S3 Fig Profile of the sialic acid on the reverse phase (RP) column. (TIF)

S4 Fig Profile of N-linked glycan on the normal phase column. (TIF)

S5 Fig Profile of the desialated N-linked glycan on the normal phase column. (TIF)

S6 Fig Profile of the desialated and degalactosilated N-linked glycan on the normal phase column. (TIF)

S7 Fig Profile of the desialated and galactosilated N-linked glycan on the normal phase column. (TIF)

S8 Fig Profile of the N-linked glycan on the weak anion exchange (WAX) column. Expected glycan charge is denoted in red. Neutral–peak of uncharged glycans, 2AB–peak of the residual reagent.
(TIF)

S9 Fig Profile of the desialated N-linked glycan on the weak anion exchange (WAX) column. Expected glycan charge is denoted in red. Neutral–peak of uncharged glycans.
(TIF)

S10 Fig Characteristic dose-response association and dissociation curves for purified FSH (kinetic analysis). BA 034220 –control FSH preparation, Gonal-f. API 110816 and API 120617 –two batches of the purified FSH from the G4 cell line.
(TIF)

S11 Fig Cell-based *in vitro* FSH activity assay dose-response curve for purified FSH. The sample (blue line) and the 2nd international standard for recombinant FSH (green line) are displayed together in each diagram.
(TIF)

S12 Fig *In vivo* FSH activity assay, mean graph and characteristic linear model regressions. Data for the purified FSH as compared with the 2nd international standard for recombinant FSH. Response–ovary mass (mg) in relation to log daily FSH dose (μg).
(TIF)

S1 Table Specific primers for sequencing, qPCR and PCR analysis of MCB gDNA. (PDF)
