# Supplementary figures and images for: High-level expression of biologically active human follicle stimulating hormone in the Chinese hamster ovary cell line by a pair of tricistronic and monocistronic vectors
Source: PLoS One. 2019 Jul 5;14(7):e0219434. doi: 10.1371/journal.pone.0219434 (PMC6611665; doi:10.1371/journal.pone.0219434)

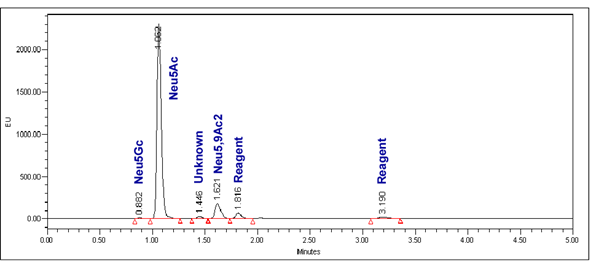

Supplement: S3 Fig — (TIF) [file pone.0219434.s003.tif]

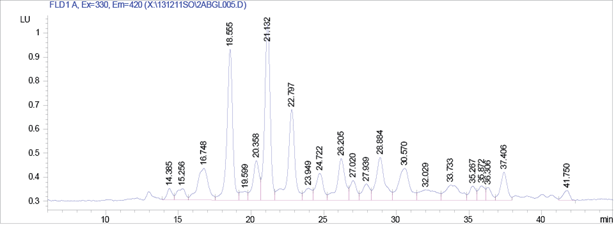

Supplement: S4 Fig — (TIF) [file pone.0219434.s004.tif]

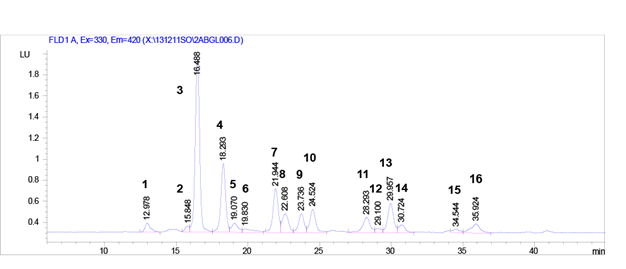

Supplement: S5 Fig — (TIF) [file pone.0219434.s005.tif]

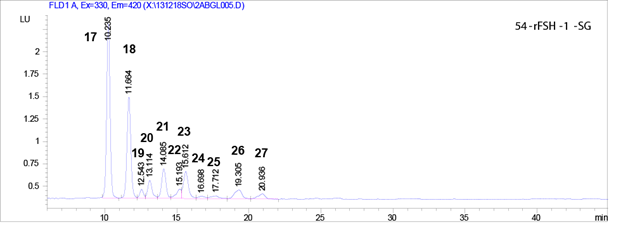

Supplement: S6 Fig — (TIF) [file pone.0219434.s006.tif]

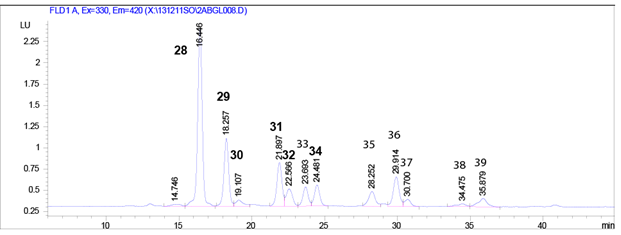

Supplement: S7 Fig — (TIF) [file pone.0219434.s007.tif]

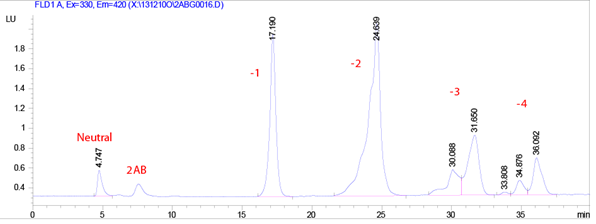

Supplement: S8 Fig — Expected glycan charge is denoted in red. Neutral–peak of uncharged glycans, 2AB–peak of the residual reagent. (TIF) [file pone.0219434.s008.tif]

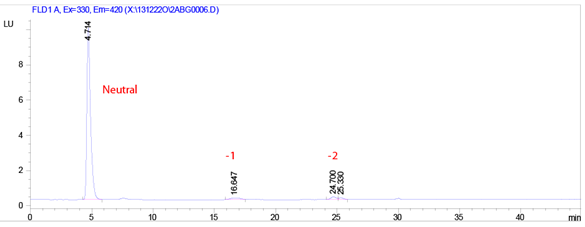

Supplement: S9 Fig — Expected glycan charge is denoted in red. Neutral–peak of uncharged glycans. (TIF) [file pone.0219434.s009.tif]

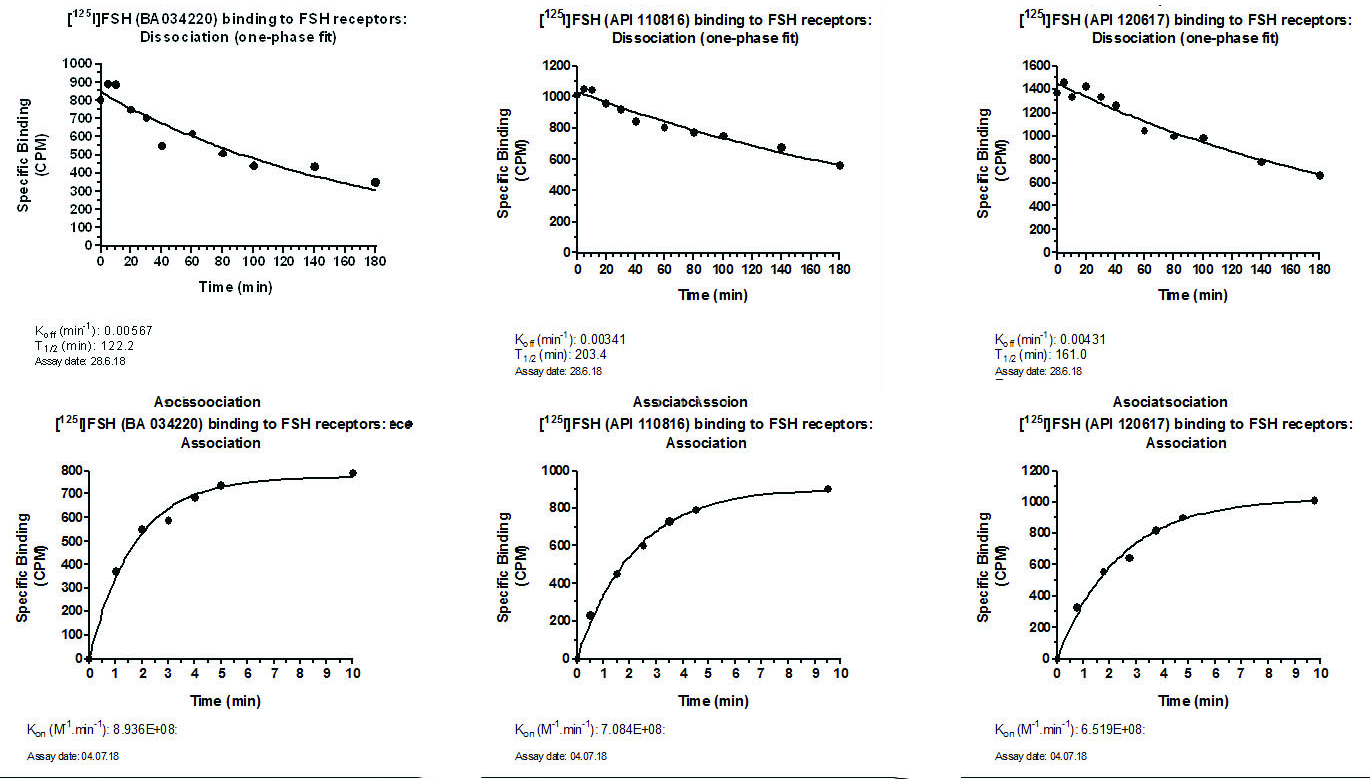

Supplement: S10 Fig — BA 034220 –control FSH preparation, Gonal-f. API 110816 and API 120617 –two batches of the purified FSH from the G4 cell line. (TIF) [file pone.0219434.s010.tif]

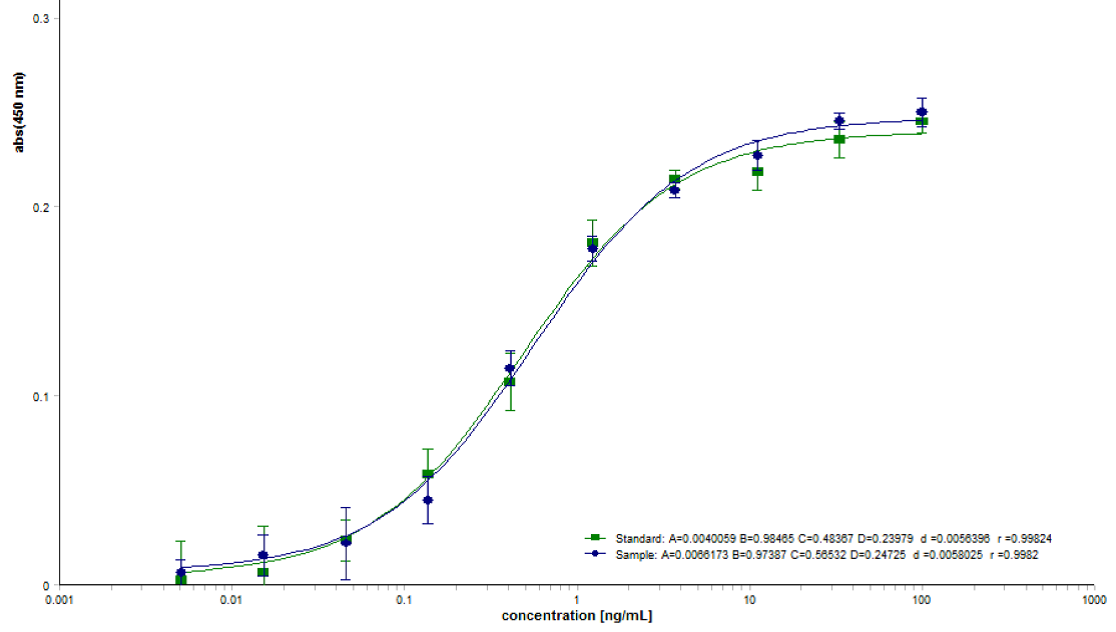

Supplement: S11 Fig — The sample (blue line) and the 2nd international standard for recombinant FSH (green line) are displayed together in each diagram. (TIF) [file pone.0219434.s011.tif]

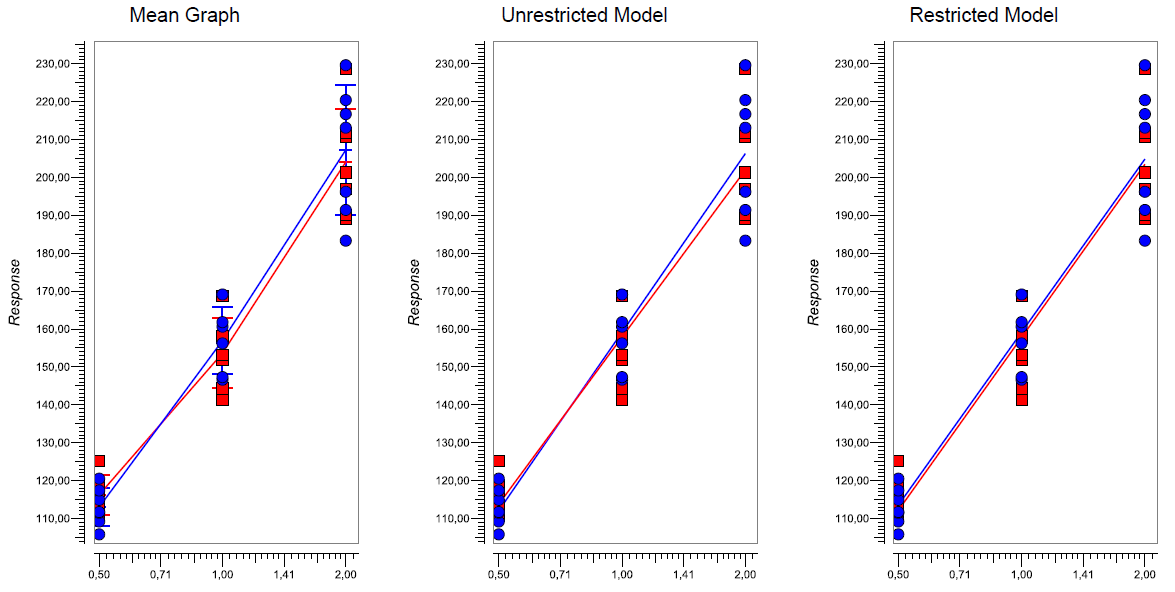

Supplement: S12 Fig — Data for the purified FSH as compared with the 2nd international standard for recombinant FSH. Response–ovary mass (mg) in relation to log daily FSH dose (μg). (TIF) [file pone.0219434.s012.tif]
