# Supplementary material for: High-level expression of biologically active human follicle stimulating hormone in the Chinese hamster ovary cell line by a pair of tricistronic and monocistronic vectors
Source: PLoS One. 2019 Jul 5;14(7):e0219434. doi: 10.1371/journal.pone.0219434 (PMC6611665; doi:10.1371/journal.pone.0219434)
Supplement: S1 Table — (PDF) [file pone.0219434.s013.pdf]

S1 Table. Specific primers for sequencing, qPCR and PCR analysis of MCB gDNA

**Name**	**Primer sequence 5' to 3'**	**Target**
SQ-5CH6-F	GCC GCT GCT TCC TGT GAC	p1.1 and p12 expression vectors
SQ-3CH1-R	ACA AAC AGT TCT GAG ACC G	
IRESA rev	AGG TTT CCG GGC CCT CAC ATT G	EMCV IRES
SQ-FA-F	CAC GCT ACA GGA AAA CCC	Human FSH alpha-chain ORF
SQ-FA-R	TCT TGG ACC TTA GTG GAG TG	
SQ-FB-F	GCC CAA AAT CCA GAA AAC	Human FSH beta-chain ORF
SQ-FB-R	ACA ATC AGT GCT GTC GCT	
RT-ID-F	GCC ACA AGA TCT GCC ACC ATG	Transgene region, including the attenuated EMCV IRES element and mouse *dhfr* gene
RT-ID-R	GTA GGT CTC CGT TCT TGC CAA TC	
RT-HYG-F	TTC GGC TCC AAC AAT GTC	Hygromycin B resistance gene from p1.2 expression vector (hygromycin B phosphotransferase)
RT-HYG-R	GTC TGC TGC TCC ATA CAA G	
RT-PPIB-F	GCA GGC AAA GAC ACC AAT G	CHO peptidyl-prolyl isomerase B (*PPIB*) gene
RT-PPIB-R	CTC CAC CTT CCT CAC TAC ATC	

F = forward primer; R = reverse primer. The RT-ID-F and RT-ID-R pair contains one primer to the IRES sequence of ECMV and the reverse primer to mouse dihydrofolate reductase gene region. Oligonucleotides were synthesized by Evrogen, JSC (Moscow, Russia).
